# Therapeutic effects of acupuncture on sensory ataxia after a cerebral hemorrhage: A case report

**DOI:** 10.1097/MD.0000000000021124

**Published:** 2020-07-17

**Authors:** Kuan-Yu Lu, Ka-Fai Yuen, Jia-Yuan Luo, Chang-Zern Hong, Li-Wei Chou

**Affiliations:** a Department of Physical Medicine and Rehabilitation, Chang Gung Memorial Hospital, Chiayi; b Department of Rehabilitation, Ton-Yen General Hospital, Tsinchu County; c Department of Physical Medicine and Rehabilitation, Puzi Hospital, Ministry of Health and Welfare, Chiayi, Taiwan; d Retired professor, Department of Physical Medicine and Rehabilitation, University of California Irvine, CA, USA; e Department of Physical Medicine and Rehabilitation, China Medical University Hospital; f Department of Physical Therapy and Graduate Institute of Rehabilitation Science, China Medical University; g Department of Rehabilitation, Asia University Hospital, Taichung, Taiwan.

**Keywords:** acupuncture, cerebral hemorrhage, rehabilitation, sensory ataxia

## Abstract

**Introduction:**

Sensory ataxia is a dysfunction of dynamic balance due to impairment of sensory input into the control of movement. The sequelae of stroke, such as hemiplegia, somatosensory impairment, and impaired balance may cause significant disability and may affect patients’ quality of life. In addition to rehabilitation programs, acupuncture therapy has been applied to stroke patients and is recommended as a complementary therapy in stroke rehabilitation.

**Patient concerns:**

A 70-year-old male had a sudden onset of conscious loss. The brain computed tomography showed intracerebral hemorrhage with subdural hemorrhage and subarachnoid hemorrhage.

**Diagnosis:**

Intracerebral hemorrhagic stroke was diagnosed.

**Interventions:**

He received craniotomy with hematoma evacuation immediately and waked up 3 weeks with bilateral hemiparesis (right side weaker than left), impaired position sensation and tactile perception in the right lower limb. He then began to receive rehabilitation therapy and had significant improvement in muscle strength and static balance, but no improvement in tactile perception of position sense in the right lower limbs and reached plateau. Then he received acupuncture therapies to *Yongquan* (KI1), *Tongtien* (BL7) and *Houxi* (SI3).

**Outcomes:**

The patient's walking ability recovered after receiving rehabilitation programs for 3 years, but the impairment in proprioception and dynamic balance persisted. The perception and dynamic balance had significantly improved after patient received acupuncture therapy, especially the acupuncture point of *Yongquan* (KI1).

**Conclusion:**

The clinical effect of acupuncture in combination with conventional rehabilitation therapy for neurological impairment recovery, improving activity of daily living performance and improving post-stroke imbalance was explored. We hope that this report can facilitate further well controlled quantitative objective studies on a big size of samples.

## 1 Introduction

Sensory ataxia is a dysfunction of dynamic balance due to impairment of sensory input into the control of movement, but not cause by cerebellar dysfunction. Sensory ataxia is distinguished from cerebellar ataxia by the presence of near-normal coordination when the movement is visually observed. However, marked worsening of coordination when the eyes are closed (a positive Romberg's sign). Sensory ataxia can be a manifestation of sensory large fiber peripheral neuropathies and conditions causing dysfunction of the dorsal columns of the spinal cord due to a variety of disorders: infectious, auto-immune, metabolic, toxic, vascular and hereditary diseases.^[1–3]^

Acupuncture (AcP) is one valuable therapeutic intervention of the Traditional Chinese medicine. According to the theory of acupuncture, there are 14 channels of energy distributed in the whole human body. Inside these 14 channels, there is “*Qi*” circulated in the whole body regularly every day. Each channel contains many acupuncture points where more “*Qi*” accumulated and being more sensitive.^[4]^ Acupuncture treatment on different acupuncture points may cure different various diseases, depending on the selection of different acupuncture points. In this case study, we applied two rules:

(1) the acupuncture points near the lesion, and
(2) the distant acupuncture points belonging the same channel passing through the lesion or the channels related to the group containing the lesion.

The lesion according to theory of acupuncture is defined as the site where symptoms and signs are present.^[5]^ A patient (a physiatrist, one of authors, CZH) with sensory ataxia due to subdural hemorrhage with sensory deficit at right sole area was selected for this case study. After therapeutic acupuncture, several subjective and objective evaluation were assessed.

## 2 Case presentation

**Figure 1:**
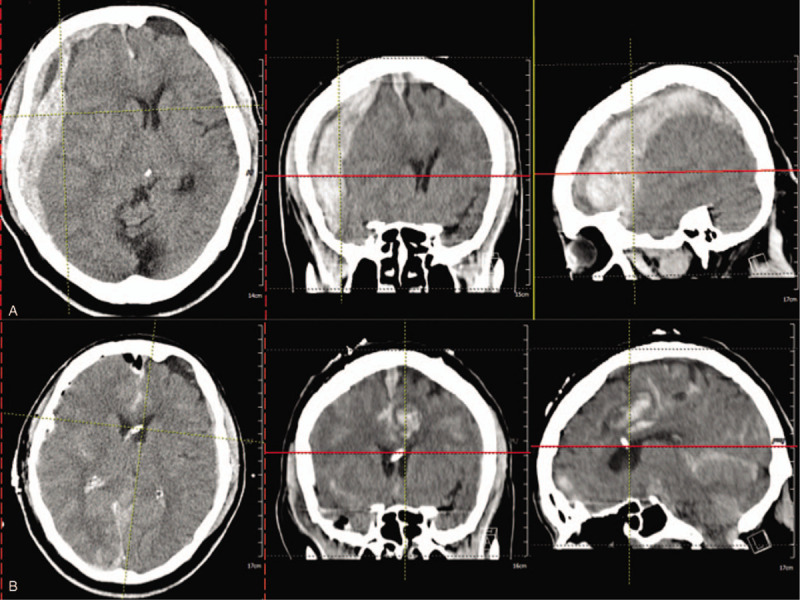
(A) Brain CT showed intra-cerebral hemorrhage with subdural hemorrhage and subarachnoid hemorrhage on 2014-12-21. (B) After craniotomy with hematoma evacuation, brain CT showed some residual hematoma still in the cerebral and cerebellar area on 2014-12-22. CT = computed tomograpghy.

A 70 year-old male physiatrist (CZH) had a sudden onset of conscious loss on 2014-12-21. He was initially brought to a teaching hospital where computed tomography showed evidence of intra-cerebral hemorrhage with subdural hemorrhage and subarachnoid hemorrhage (Fig. 1A). He then received craniotomy with hematoma evacuation immediately (Fig. 1B). He waked up 3 weeks after craniotomy but found to have bilateral hemiparesis (right side weaker than left) with impaired position sensation and tactile perception in the right lower limb. He then began to receive rehabilitation therapy since after waked up. He discharged to a nursing home about 3 months later to continued rehabilitation therapy. He had significant improvement in muscle strength and static balance. He was then able to control wheelchair by himself by 2015-02. Gradually, he was able to walk with a walker by 2016-01, then a cane by 2016-05, but still had impaired dynamic balance. There was no improvement in tactile perception of position sense in the right lower limbs.

On 2017-12-30, he received acupuncture therapy to *Yongquan* (KI1). Size 36 acupuncture needles with 1 inch in length were inserted into the acupuncture points in sequence. Bilateral *Yongquan* (KI1) (in the kidney channel) were selected for near acupuncture point, and bilateral *Tongtien* (BL7) (in the bladder channel) and bilateral *Houxi* (SI3) (in the small intestine channel) for distant acupuncture point.^[6]^ After each needling, electric stimulation with frequency of 2 Hz were applied for 30 minutes for each point. After only 1 treatment of acupuncture, he had significant improvement in perception, and dynamic balance.

The case study was conducted after the first acupuncture therapy since the patient found the dramatic therapeutic effectiveness of acupuncture. The findings of evaluation before acupuncture was estimated by the patient depending on his memory. After therapeutic acupuncture, the following items were assessed:

(1) Subjective feeling of dynamic stability (dynamic balance during walking) (Sd);
(2) Subjective feeling of motor function (strength, static balance) (Sm);
(3) Subjective feeling of sensory function (perception of position, tactile perception) (Ss);
(4) Objective evaluation of walking speed (Ws);
(5) Objective evaluation of walking tolerance (Wt);
(6) Objective evaluation of static stability, 2 LE standing time with eye closed (2LE);
(7) Objective evaluation of static stability, that is, the duration of 1-leg standing time with eye open (1LE). The scales of assessment are listed in Table 1.

**Table 1:**
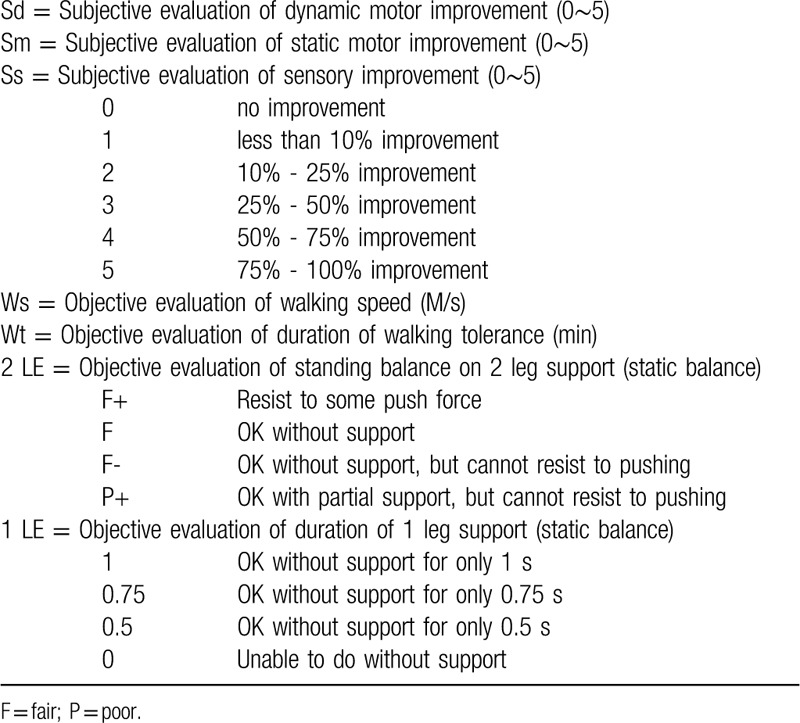
The scales of assessment.

The results of assessment of functional improvement are listed in Table 2. The first row of the table showed the procedure (B = before AcP; R = resting period before the next AcP). The 2nd row listed the date of AcP, and the 3rd row showed the duration of AcP (30 minutes each AcP treatment, or the days between 2 AcP treatments. After the 4th to 10th rows are the functional improvement as explained in the legends of the table.

**Table 2:**
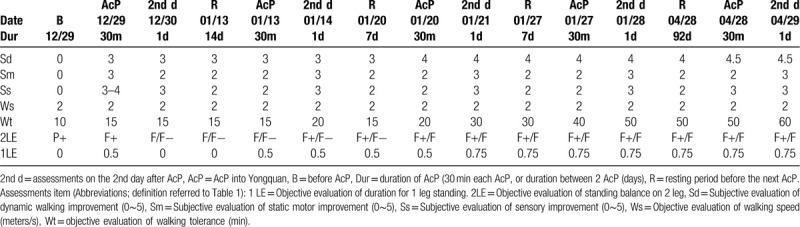
Assessment of functional improvement.

The most remarkable findings include the subjective walking (dynamic balance) and objective assessment of 1 leg standing balance (static balance). It appears that the subjective feelings of both motor improvement and to that of sensory improvement were both remarkable immediately after AcP treatment. But they were not sustained, but declined. Walking speed remained unchanged throughout the whole studying period. Walking tolerance improved after each AcP treatment, even in the last treatment. Regarding the objective balance, only little improvement in the 2 leg supported balance, but remarkable improvement in the 1 leg standing balance and became stable after 3 treatments of AcP.

## 3 Discussion

All the assessments in this case study were improved except for walking speed. It appeared that these improvements were related to the effectiveness of acupuncture. The patient was considered to reach a “plateau” of rehabilitation, until the physician applied the acupuncture to the point of *Yongquan* (KI1) to the patient. *Yongquan* (KI1) can treat conscious disturbance, cognitive function impairment, sole unable to touch the ground.^[7]^ Quantitative and accurate measurements are crucial to evaluate the treatment outcomes. The most important findings in this case study include the remarkable improvement in the subjective assessment of dynamic balance (walking) and the objective assessment in 1 leg standing, an important indicator of static balance. Both improvement had continuous improvement even after cessation of acupuncture.

In addition to subjective sensations of motor and sensory improvement, we also use several objective tests to evaluate the improvements after *Yongquan* (KI1) acupuncture treatment, including walking speed, walking tolerance and the standing stability with 1 leg and 2 legs support. The overall results indicate the positive effect of acupuncture treatment with *Yongquan* (KI1) except the walking speed. The walking speed remains a steady performance. According to the patient, the subjective feelings of walking balance (dynamic balance) was remarkable, unfortunately we did not design a quantitative and objective measurement of dynamic balance. The subjective feelings of motor and sensory improvement are both also remarkable and immediate increased after acupuncture. Though the effects declined gradually as time goes by, but compare with the baseline data on 2017-12-29, the improvement of subjective feelings of motor and sensory is still marked.

Another important finding is the improvements in the walking tolerance. According to the above data, the patient could only walk without assistance for 10 minutes initially, but the walking time gained gradually with treatment. After complete the whole acupuncture treatment course, the patient can tolerate 60 minutes walking without assistance. Regarding to the dynamic balance, standing with 2 legs and 1 leg supported are chosen to evaluate the treatment results. The improvements of dynamic balance are observed after acupuncture with *Yongquan* (KI1), especially 1 leg standing balance. Though the patient can stand with 2 legs without assistance at initial assessment, he cannot resist to the pushing. After treatment, the patient feels subjectively that there is only little improvement in the 2 legs supported balance, but able to resist pushing force is noticed. As for the 1 leg standing balance, the patient can barely stand with 1 leg (0 sec) when first evaluate on 2017-12-29. After treatment, the duration of standing with 1 leg support can lasting for 0.75 sec. It's worth noting that, the improvement of dynamic balance can be sustained after treatment without declined. Lower limbs somatosensory impairment has negative effects on walking tolerance and dynamic balance, etc. Whereas those factors: walking tolerance and dynamic balance play important roles of ambulation and are correlated with the activity of daily living (ADL) performance. Thus, when the patient's foot sensory input regained after acupuncture therapy with *Yongquan* (KI1), the functional performance also improved.

The sequelae of stroke, such as hemiplegia, somatosensory impairment, and impaired balance may cause significant disability and may affect patients’ quality of life.^[8–10]^ Though patients may regain the ability to walk after undergoing rehabilitation, but still have higher risk to accidental falls due to impaired balance.^[9,11]^ Balance deficits may also affect ADL functions such as transfers, ambulation, bath room activity, and so on. Balance can be influenced by many factors including motor function, sensory function, vestibular function, visual function, etc.^[11–13]^ However, patients with sensory impairment seems tend to have worse functional recovery.^[14]^ In this case, the patient's walking ability recovered after receiving rehabilitation programs for 3 years, but the impairment in proprioception and dynamic balance persisted. The perception and dynamic balance had significantly improved after patient received acupuncture therapy, especially the acupuncture point of *Yongquan* (KI1).

Previous reports about the prevalence of somatosensory deficits in chronic stoke patents varied in a wide range which probably related to the inadequate precise standard measurements of somatosensory deficits.^[15–17]^ However, some authors believed that, somatosensory impairments are common in chronic stroke patients and which may influence the functional performance.^[15,17]^ In chronic stroke patients, rehabilitation is often terminated when additional improvement has been reached “plateau” which may occur 6 months later after stroke.^[17–19]^ However, the exact recovery course of somatosensory impairments is still not well documented, also, there are still no strong evidences can proof that the therapeutics of rehabilitation can effectively improve the somatosensory deficits.^[17,20]^

In addition to rehabilitation programs, acupuncture therapy has been applied to stroke patients and is recommended as a complementary therapy in stroke rehabilitation.^[8,21–24]^ The clinical effect of acupuncture in combination with conventional rehabilitation therapy for neurological impairment recovery, post-stroke shoulder pain, aphasia, improving ADL performance and improving post-stroke imbalance was explored.^[8,21–26]^ Some researchers reported evidences of increase cerebral blood flow in sensorimotor area and activation of somatosensory cortex in stroke patients after acupuncture therapy, by using studies with images, single photon emission computed tomography (SPECT) perfusion and functional MRI to illustrate. Those studies demonstrated the therapeutic effect of acupuncture in stroke rehabilitation.^[11,14,27,28]^

According to the theory of acupuncture in Traditional Chinese Medicine, acupuncture can direct *Qi* to the site that is lacking *Qi*, enhance the restoration of deficits.^[4]^ Previous studies have indicated that acupuncture therapy is effective in improving balance function.^[8,11,13,28,29]^ In some cases, the effectiveness can occur immediately after acupuncture. It also can affects the sensory perceptions. The selection of acupuncture points usually depends on the physicians’ personal experience in clinical practice, or the presenting symptoms of each individual patient.^[22]^ Acupuncture points, *Baihui* (GV20), *Yanglingquan* (GB34) and *Zusanli* (ST36) are often chosen for treating the impairment of balance or coordination in stroke patients.^[8,21,28,30]^ As for *Yongquan* (KI1), related studies are few,^[30]^ but in ancient Chinese medicine theory, *Yongquan* (KI1) can treat conscious disturbance, cognitive function impairment, sole unable to touch the ground.^[7]^ In a recent study conducted by Zhang's colleagues,^[31]^ they found that acupuncture at the *Yongquan* acupoints may induced stronger neuronal activity and increase synaptic activity in some areas of the brain. Long-term acupuncture may affect the quantity and function of synapses in brain area, leading the neural reorganization.

## 4 Conclusion

This is just a single case study, and hopefully this study can facilitate more studies in this aspect. From this case, we have observed the effects and benefits of treating chronic stroke patient with *Yongquan* (KI1). From the pilot case study, more well-designed investigations may be performed in the future. Furthermore, precise quantitative and objective measurements to evaluate treatment outcomes should be designed in the future studies.

## Author contributions

**Methodology:** Chang-Zern Hong.

**Supervision:** Li-Wei Chou.

**Writing – original draft:** Kuan-Yu Lu, Ka-Fai Yuen, Jia-Yuan Luo.

**Writing – review & editing:** Chang-Zern Hong, Li-Wei Chou.
